# Long‐Term Cost Utility Analysis of Endoscopic Sinus Surgery: Analysis of 5‐Year Outcomes

**DOI:** 10.1002/lary.70176

**Published:** 2025-10-04

**Authors:** Dara R. Adams, Katherine Tashman, Eric H. Holbrook, Stacey T. Gray, Ralph Metson, George Scangas

**Affiliations:** ^1^ Department of Otolaryngology—Head and Neck Surgery University of Illinois Chicago College of Medicine Chicago Illinois USA; ^2^ Department of Otolaryngology—Head and Neck Surgery Massachusetts Eye and Ear, Harvard Medical School Boston Massachusetts USA

**Keywords:** cost utility analysis, endoscopic sinus surgery, surgical outcomes

## Abstract

**Objective:**

Both endoscopic sinus surgery (ESS) and medical management have shown effectiveness for treatment of chronic rhinosinusitis (CRS); however, the majority of such analyses have relied on short‐term surgical outcomes. We aimed to evaluate the long‐term (5‐year) cost‐effectiveness of ESS versus medical management for patients with CRS.

**Methods:**

A cohort‐style Markov decision‐tree economic model with a 33‐year time horizon was developed. A cohort of 96 CRS patients who underwent ESS were compared with a 2 to 1 matched cohort of 48 CRS patients who were treated with medical management at the same academic medical center. Long‐term utility scores were calculated from the EuroQol 5‐Dimension (EQ‐5D) survey at 5‐year follow up for the surgical cohort and 1–2 year follow up for the medical management cohort. Decision‐tree analysis and a 10‐state Markov model utilized published event probabilities and primary data to calculate long‐term costs and utility. The primary outcome measure was the incremental cost‐effectiveness ratio (ICER), which represents incremental cost per quality‐adjusted life year (QALY).

**Results:**

The ESS strategy cost more ($63,296.10) but yielded increased QALYs (22.61). In comparison, the medical management strategy cost $26,990.27 but yielded only 13.48 QALYs. The ICER for ESS versus medical therapy alone was $4367.68 per QALY.

**Conclusion:**

This study shows ESS to be a cost‐effective intervention compared to medical therapy alone for the management of patients with CRS based on analysis evaluating long‐term (5‐year) surgical outcomes.

**Level of Evidence:**

N/A.

## Graphical abstract

This study utilizes a cohort‐style Markov decision tree economic model to evaluate the long‐term (5‐year) cost‐effectiveness of endoscopic sinus surgery versus medical management for patients with chronic rhinosinusitis. The results show that the endoscopic sinus surgery strategy cost more ($63,296.27) but yielded increased quality‐adjusted life years (QALYs; 22.61) compared to medical management ($26,990.27, 13.48 QALYs), signifying an incremental cost‐effectiveness ratio of $4367.68 per QALY. Therefore, endoscopic sinus surgery is a cost‐effective intervention compared to medical therapy alone for the management of patients with chronic rhinosinusitis.

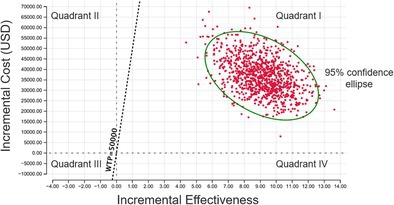

## 1 Introduction

Chronic rhinosinusitis (CRS) is a common inflammatory sinonasal disease that affects approximately 5%–10% of the US population and has a significant impact on quality of life and productivity [1, 2, 3, 4]. Indeed, patients with CRS have been found to score similarly on patient‐reported outcome measures (PROMs) as patients with diabetes mellitus, certain neoplasms, HIV, chronic obstructive pulmonary disease, and asthma [5, 6]. Furthermore, the economic burden of CRS is substantial, with estimated annual direct costs of $10–13 billion [1, 3, 7, 8, 9] and indirect costs of $13–$20 billion [4, 9].

The accepted treatment paradigm for CRS begins with appropriate medical management often consisting of saline irrigations and intranasal steroids, supplemented by antibiotics and/or systemic steroids depending upon the clinical presentation. Additional medications to treat contributing inflammatory and allergic factors may also be utilized. In recent years, biologics have been introduced for the medical treatment of patients with CRS with nasal polyps. These monoclonal antibody preparations have been shown to reduce polyp burden in this patient cohort. For CRS patients who fail appropriate medical management, endoscopic sinus surgery (ESS) is the mainstay of treatment.

Previous economic models have found ESS to be cost‐effective when compared to medical therapy alone for patients with CRS with and without nasal polyps [10, 11, 12]. However, these models have relied on relatively short‐term (2 years or less) surgical data with extrapolated long‐term outcomes. Additionally, the medical cohort in the majority of these analyses has been managed by primary care physicians and not by otolaryngologists [10, 11].

The aim of the current study was to improve upon prior economic models to evaluate the cost‐effectiveness of ESS compared to continued medical therapy by including long‐term (5‐year) surgical outcomes data and by utilizing prospectively collected utility data on patients medically managed by an otolaryngologist. The authors hypothesize that ESS remains a cost‐effective intervention for patients with CRS in this robust economic model.

## 2 Materials and Methods

### 2.1 Economic Model and Study Cohorts

Patients with CRS were tracked through two treatment pathways within a decision tree and 10‐state Markov microsimulation model: continuous medical treatment or ESS and postoperative medical management. This analysis updates prior models from our research group [10, 11, 13] with several key improvements, including (1) the use of prospectively gathered data among patients medically managed by an otolaryngologist in the medical arm; (2) the inclusion of 5‐year effectiveness outcomes for the surgical arm; and (3) updated cost benchmarks. Per *The Laryngoscope's* guidelines, the details of the prior models are not repeated in this manuscript and are available by reading our prior publications. Only methodological changes from the prior analyses are highlighted below. This manuscript follows the 2013 Consolidated Health Economic Evaluation Reporting Standards guidelines. This study was approved by the institutional review board of Mass General Brigham. Informed consent was obtained from all participants.

The medical arm was recruited from the practices of two rhinologists at the Massachusetts Eye and Ear between April 2015 and March 2017 and consisted of 48 new CRS patients treated with appropriate medical management. None of these patients were treated surgically prior to or during the study period. Treatments prescribed during the study period included saline irrigations (79.2%), nasal steroid sprays (81.3%), topical and oral antihistamines (47.9%), nasal steroid irrigations (29.2%), and oral antibiotics (33.3%). There were no patients treated with biologics for their sinonasal disease. The inclusion/exclusion criteria as well as methods of data collection for this cohort have been previously described [14]. Patients were followed prospectively and completed the EuroQol 5‐Dimension (EQ‐5D) survey at the time of enrollment and annually for 2 years. Medical cohort patients were entered directly into a 10‐state Markov model, described in detail below.

The medical cohort was matched 2 to 1 with the surgical cohort with the standardized mean differences for age, sex, race, ethnicity, smoking status, and nasal polyposis all less than 0.1. Matching was performed in R 4.2.3 using optimal full matching with a 2 to 1 ratio in the MatchIt package [15, 16]. Matching demographics were specifically chosen as they have been found to impact health utility values (HUV) in an independent analysis evaluating patients with medically treated CRS [14]. Approximately one‐quarter of patients in the medical (27.1%) and surgical (29.2%) cohorts had nasal polyps.

There were 96 CRS patients in the surgical cohort. The methods of data collection have been previously described by the authors [10, 11, 13, 17, 18, 19]. Patients received post‐operative medical management at the discretion of their surgeon. Patients were followed prospectively and completed the EQ‐5D survey at the time of surgery and annually for 5 years.

**FIGURE 1:**
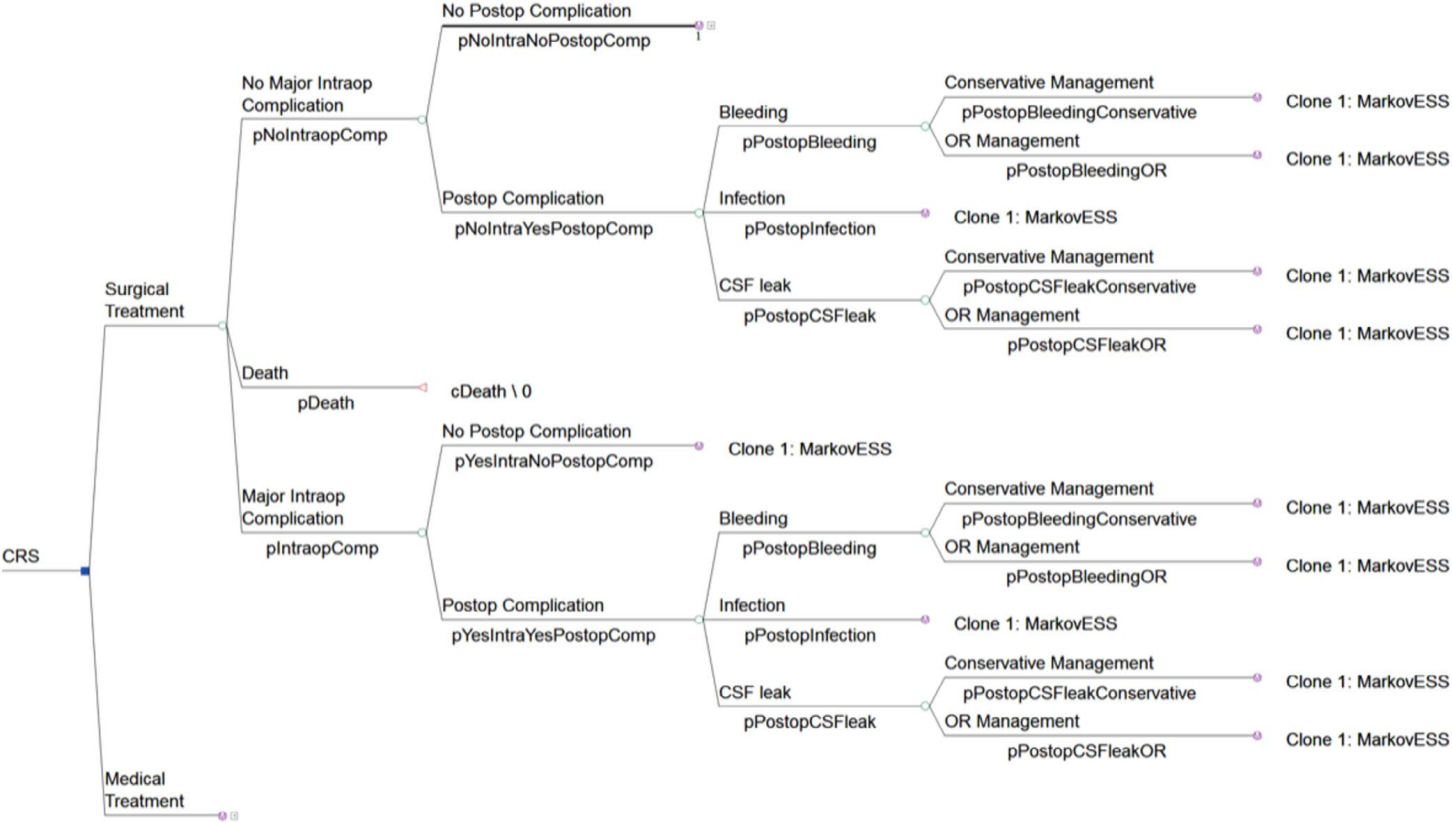
Decision tree for intraoperative and postoperative outcomes for ESS. All terminal nodes (aside from death) are represented as Markov models. CRS = chronic rhinosinusitis, CSF = cerebrospinal fluid, OR = operating room, Postop = postoperative.

All patients traversed a perioperative decision tree (Figure 1) which details possible intraoperative and postoperative complications.

### 2.2 Effectiveness

Health state utility data was obtained for each patient using the EQ‐5D at 5‐year follow up for the surgical cohort and 1–2 year follow up for the medical cohort. Although this discrepancy could impact the study results, the study design was made with the understanding that most patients in the medical cohort had been treated for their sinonasal disease for many years prior to enrollment, as is typical for patients with CRS. The EQ‐5D is a general health‐related quality‐of‐life instrument that collects information from five health domains: mobility, self‐care, usual activity, pain/discomfort, and anxiety/depression [17]. Previous studies by our group have shown that disease specific survey scores for CRS (22‐item Sino‐Nasal Outcome Test, SNOT‐22) correlate with EQ‐5D scores, indicating construct validity of the EQ‐5D in CRS [18, 19]. HUVs were calculated from the EQ‐5D utilizing a published algorithm.

### 2.3 Probabilities

Postoperative complication rates for ESS were obtained from a PUBMED literature search previously published and described by this group [10]. Probabilities and sources are presented in Table 1.

**TABLE 1:** Description of model parameters used in the economic model.

Variable	Description	Mean value	Standard error	Source
*Probability*				
pDeath	Probability of death from ESS	0.0001	0.0003	Lund et al. [20]
pIntraOpComp	Probability of intraoperative complication	0.0095	0.0004	May et al. [21] Ramakrishnan et al. [22]
pNoIntraOpComp	Probability of no intraoperative complication	0.9904	0.0004	Ramakrishnan et al. [22]
pNoPostOpComp	Probability of no postoperative complication	0.8273	0.0069	Dalziel et al. [23] Rudmik et al. [12]
pPostOpComp	Probability of postoperative complication	0.1727	0.0069	Dalziel et al. [23] Rudmik et al. [12]
pPostOpInfection	Probability of postoperative infection (assuming a postoperative complication occurred)	0.647	0.047	Dalziel et al. [23] Rudmik et al. [12] May et al. [21]
pPostOpEpistaxis	Probability of postoperative epistaxis (assuming a postoperative complication occurred)	0.352	0.047	May et al. [21] Stankiewicz et al. [24]
pPostOpCSFleak	Probability of postoperative CSF leak (assuming a postoperative complication occurred)	0.001	0.0002	Ramakrishnan et al. [22]
pPostOpEpistaxisCons	Probability of epistaxis resolving with conservative management	0.27	0.069	Stankiewicz et al. [24]
pPostOpEpistaxisOR	Probability of epistaxis requiring OR management	0.73	0.069	Stankiewicz et al. [24]
pPostOpCSFleak Cons	Probability of CSF leak resolving with conservative management	0.19	0.054	Lindstrom et al. [25]
pPostOpCSFleak OR	Probability of CSF leak requiring OR management	0.81	0.054	Lindstrom et al. [25]
pRevisionESS	Probability of revision ESS/year	0.041	0.005	Hopkins et al. [26]
*Cost*				
cDeath	Cost of death	$22,548		Kramer et al. [27]
cESS	Cost of ESS	$10,500		Orlandi et al. [28]
cMedical	Cost of medical treatment for CRS	$772		Rudmik et al. [3]
cESSMajorComp	Cost of major complication	$8354		Ge et al. [29]
cInfection	Cost of infection	$559		Rudmik et al. [12]
cBleedCons	Cost of minor epistaxis	$599		Nithianandan et al. [30]
cBleedOR	Cost of major epistaxis	$6450		Dedhia et al. [31]
cCSFCons	Cost of self‐limited CSF leak	$13,594		Rudmik et al. [12]
cCSFOR	Cost of major CSF leak	$15,818		Johnson et al. [32]
cMarkov1	Cost of Markov health state #1	$1167	$1167	
cMarkov2	Cost of Markov health state #2	$1408	$1408	
cMarkov3	Cost of Markov health state #3	$1649	$1649	
cMarkov4	Cost of Markov health state #4	$1890	$1890	
cMarkov5	Cost of Markov health state #5	$2131	$2131	
cMarkov6	Cost of Markov health state #6	$2372	$2372	
cMarkov7	Cost of Markov health state #7	$2613	$2613	
cMarkov8	Cost of Markov health state #8	$2854	$2854	
cMarkov9	Cost of Markov health state #9	$3095	$3095	
cMarkov10	Cost of Markov health state #10	$3336	$3336	

The Markov model analyzed 10 health states in order to rigorously reflect the possible range of disease states among patients with CRS [33, 34, 35]. Transition probabilities were calculated from the primary data of the cohorts and are shown in Table 2 (Medical, entrance and first year transition probabilities) and Table 3 (Surgical, entrance, first year, and second year transition probabilities). The Markov model used the first year transition probability in Cycle 1 and first to fifth year transition probabilities, calculated from all patients who returned completed Year 1 and Year 5 surveys, for all other cycles. All medical treatment patients were assumed to not undergo surgery during the time course of the study.

**TABLE 2:** Transition probabilities for the treatment years between all possible Markov health states for the medical cohort.

		Year 1									
		0–0.099	0.1–0.199	0.2–0.299	0.3–0.399	0.4–0.499	0.5–0.599	0.6–0.699	0.7–0.799	0.8–0.899	0.9–1
Year 0	0–0.099	0	0	0	0	0	0	0	0	0	0
	0.1–0.199	0	0	0	0	0	0	0	0	0	0
	0.2–0.299	0	0	0	0	0	0	0	0	0	0
	0.3–0.399	0	0	0	0	0	0	0	0	0	0
	0.4–0.499	0	0	0	0	0	0.25	0.25	0	0.25	0.25
	0.5–0.599	0	0	0	0	0	0	0	0	0	0
	0.6–0.699	0	0	0	0	0	0	0	0.17	0.17	0.67
	0.7–0.799	0	0	0	0	0	0	0	0.25	0.25	0.5
	0.8–0.899	0	0	0	0	0	0	0.03	0.03	0.22	0.72
	0.9–1	0	0	0	0	0	0	0	0.024	0.12	0.86

**TABLE 3:** Transition probabilities for the first and second postoperative years between all possible Markov health states for the surgical cohort.

		Year 1									
		0–0.099	0.1–0.199	0.2–0.299	0.3–0.399	0.4–0.499	0.5–0.599	0.6–0.699	0.7–0.799	0.8–0.899	0.9–1
Year 0	0–0.099	0	0	0	0	0	0	0	0	0	0
	0.1–0.199	0	0	0	0	0	0	0	0	0	0
	0.2–0.299	0	0	0	0	0	0	0	0	0	0
	0.3–0.399	0	0	0	0	0	0	0	0	0	0
	0.4–0.499	0	0	0	0	0	0.25	0.25	0	0.25	0.25
	0.5–0.599	0	0	0	0	0	0	0	0	0	0
	0.6–0.699	0	0	0	0	0	0	0	0.17	0.17	0.67
	0.7–0.799	0	0	0	0	0	0	0	0.25	0.25	0.5
	0.8–0.899	0	0	0	0	0	0	0.03	0.03	0.22	0.72
	0.9–1	0	0	0	0	0	0	0	0.024	0.12	0.86

		Year 2									
		0–0.099	0.1–0.199	0.2–0.299	0.3–0.399	0.4–0.499	0.5–0.599	0.6–0.699	0.7–0.799	0.8–0.899	0.9–1
Year 1	0–0.099	0	0	0	0	0	0	0	0	0	0
	0.1–0.199	0	0	0	0	0	0	0	0	0	0
	0.2–0.299	0	0	0	0	0	0	0	0	0	0
	0.3–0.399	0	0	0	0	0	0	0	0	0	0
	0.4–0.499	0	0	0	0	0	0	0	0	0	0
	0.5–0.599	0	0	0	0	0	0	0	0	0	0
	0.6–0.699	0	0	0.33	0	0	0	0	0.67	0	0
	0.7–0.799	0	0	0	0	0	0	0.5	0	0.25	0.25
	0.8–0.899	0	0	0	0	0	0	0.24	0.1*8*	0.24	0.35
	0.9–1	0	0	0	0	0	0	0.04	0.04	0.19	0.72

### 2.4 Cost

A mean total cost of uncomplicated ESS of $10,500 was reported in the International Consensus Statement on Allergy and Rhinology: Rhinosinusitis 2021, while the incremental cost of ESS with major intraoperative complication was reported to be $8354 [29]. Therefore, this cost was assigned to all major intraoperative complications. The cost of various postoperative complications was obtained from the literature, and all costs have been updated from our group's previous models for this analysis (Table 1).

### 2.5 Sensitivity Analysis

**FIGURE 2:**
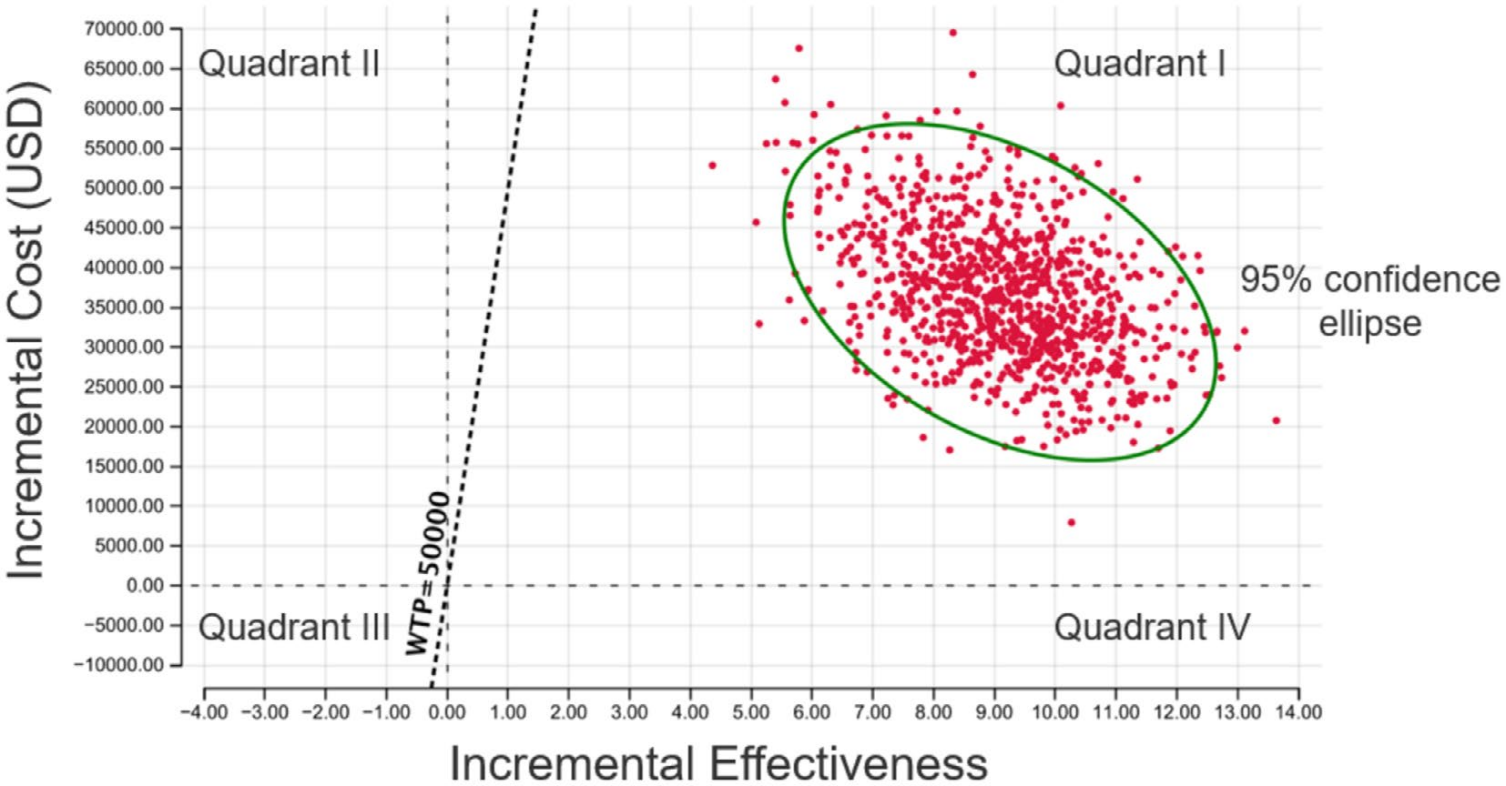
The ICER scatter plot depicts all ICERs generated from the Monte Carlo simulation onto the cost‐effectiveness plane. In this study, greater than 99% of individual ICER outcomes for surgical vs. medical treatment were in quadrant I (greater utility than medical treatment and below the $50,000‐per‐QALY threshold). HUV = health utility value, ICER = incremental cost‐effectiveness ratio, QALY = quality‐adjusted life year.

**FIGURE 3:**
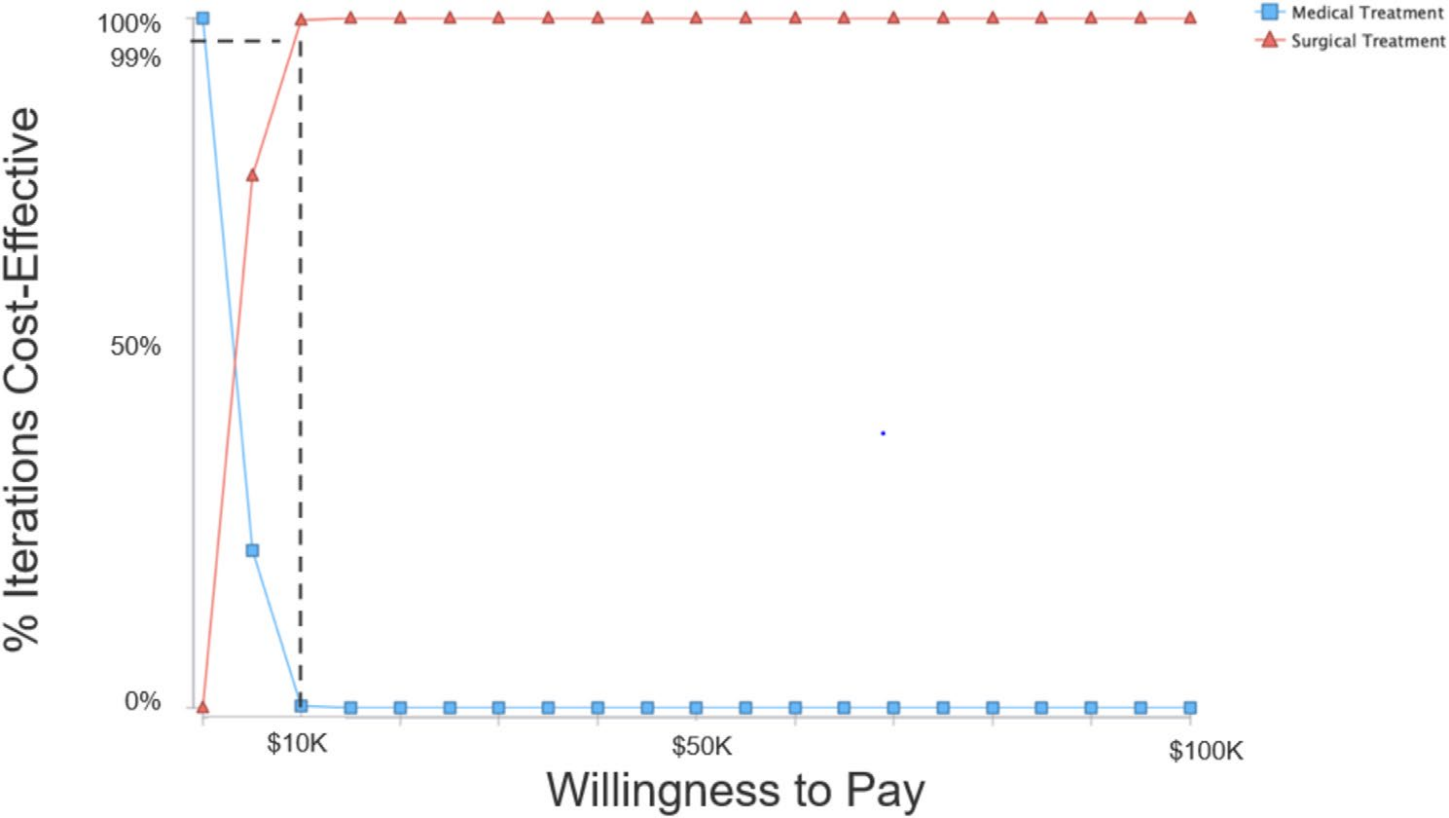
The cost‐effectiveness acceptability curve depicts the degree of certainty that a particular treatment option is the most cost‐effective option. This study demonstrates a $10,000 threshold above which the surgical treatment option is more cost‐effective with 99% certainty or greater.

A Monte Carlo microsimulation with 10,000 samples was executed. The main outcome of this analysis was a comparison of the cost effectiveness of ESS versus medical management, which is conventionally expressed as by the incremental cost‐effectiveness ratio (ICER). The ICER is calculated by dividing the difference in the cost of two strategies by the difference in the effectiveness of the same two strategies. An ICER scatter plot (Figure 2) and a cost‐effectiveness acceptability curve (CEAC, Figure 3) were created [36, 37]. The ICER scatter plot demonstrates where each ICER generated from the Monte Carlo simulation falls in terms of cost (y‐axis) and effectiveness (x‐axis). It is divided into four quadrants, including Quadrant IV (dominant choice, where ICERs are both cheaper and more effective), and Quadrant II (dominated choice, where ICERs are both more expensive and less effective). Quadrant I is where ICERs that are more expensive yet more effective fall, and the decision to select this intervention depends on the willingness‐to‐pay (WTP) threshold, which is the maximum ICER that policy makers are willing to pay [38]. Historically, $50,000 per QALY is a commonly used benchmark in cost utility analyses performed since the 1990s and was used as a conservative choice in this analysis; however, recent studies have suggested that this value may represent a low benchmark in the current U.S. healthcare system [39]. The CEAC represents the probability that a strategy is optimal for a range of WTP values and is critical to understand when drawing conclusions on the cost effectiveness of a strategy.

## 3 Results

### 3.1 Cost Effectiveness

The ESS strategy cost a total of $63,296.10 and produced 22.61 quality‐adjusted life years (QALYs). The medical‐therapy‐alone strategy cost a total of $26,990.27 and produced 13.48 QALYs. The ICER for ESS versus medical therapy alone is $4367.68 per QALY.

### 3.2 Sensitivity Analysis: One‐Way Sensitivity Analysis, Cost of Surgery

A one‐way sensitivity analysis was conducted on cost of surgery, which was varied from $10,500 to $21,000 per ESS. The results demonstrate a range of ICERs from $4367.68 to $6857.59 per QALY, respectively.

### 3.3 Sensitivity Analysis: Probabilistic Sensitivity Analysis

The ICER scatter plot (Figure 2) demonstrates that, at a WTP threshold of $50,000/QALY, in 99% of individual ICER outcomes, surgical treatment was more cost‐effective than medical treatment. The CEAC (Figure 3) demonstrates that, above a WTP threshold of $10,000, surgical treatment is more cost‐effective with 99% certainty or greater.

## 4 Discussion

It is well known that healthcare expenditure in the United States exceeds that of many other countries, yet outcomes are not necessarily improved. As the national cost of healthcare continues to rise, understanding the cost‐effectiveness of different interventions has taken on an increasing urgency. This need is particularly important for prevalent, chronic diseases, such as CRS, that profoundly impact patient quality of life while sustaining significant direct and indirect economic costs. Indeed, the rising prominence of patient‐reported outcomes measures (PROM) in both research and clinical practice within rhinology over the last decade is evidence of this trend [40].

Decision tree and Markov modeling is a type of economic analysis which allows for the tabulation of accumulated costs and benefits of various treatment options for chronic diseases [41]. To compare two different management strategies, the ICER is calculated by dividing the incremental cost of a new intervention by the incremental change in effectiveness [41]. The lower the ICER between two treatment options, the more cost‐effective (better value) the intervention becomes from a policy maker's perspective. By comparing an ICER to pre‐defined thresholds of cost‐effectiveness for a healthcare system, policy makers can better evaluate the value of the care that is being provided to patients.

The current cost‐utility analysis demonstrating the economic benefits of surgical versus medical treatment for CRS patients yielded an ICER of $4367.68 per QALY. In comparison, a prior study with two‐year postoperative follow up by Scangas et al. [10] yielded an ICER of $5687.41 for patients with CRS with nasal polyps and $5405.44 for those CRS without nasal polyps. The improvement in the cost‐effectiveness of ESS in the current study is consistent with prior studies even with the inclusion of longer‐term (5‐year) postoperative utility data into the Markov model. In addition, by utilizing a recently published, prospectively collected utility dataset of medically treated CRS patients, the authors aimed to provide a more accurate comparison of long‐term surgical and medical management in CRS patients who present for otolaryngologic care [14].

To our knowledge, the only other cost utility analysis that utilized primary data for both surgical and medical treatment of CRS was developed by Rudmik et al. [4, 9, 12]. This analysis identified an ICER of $5901.90 per QALY. The authors measured utility values with the Short‐Form Six‐Dimension (SF‐6D), a general health‐related quality of life PROM. Surgical outcomes were evaluated among a cohort of 168 patients at 1.5 years post operatively, and medical outcomes were obtained from the same prospective cohort. Aside from the short time course, patients cycled between only 3 health states, which may have limited the capture of more granular positive and negative HUV changes in a long‐term cost utility analysis.

The finding that the ICER of surgical management improves with the inclusion of longer‐term outcomes data is not surprising given that gains in quality of life after sinus surgery have been shown to be durable over long‐term follow‐up. Indeed, measurement of outcomes after sinus surgery utilizing both the rhinology‐specific SNOT‐22 and the general health‐related EQ‐5D shows improvement in scores after surgery that persist at 5 years postoperatively [5, 42]. Improvements in quality of life are accompanied by decreased direct costs, such as prescription medical therapy and outpatient physician visits, as demonstrated by a recent medical claims study which found that patients who underwent ESS filled fewer prescriptions across all categories (antihistamines, antibiotics, anticholinergics, decongestants, steroids, leukotriene modifiers) post‐operatively compared to pre‐operatively [43]. Furthermore, patients had fewer physician visits in the 6 months following surgery compared to the 6 months preceding surgery. Other studies comparing healthcare utilization before and after ESS have confirmed a reduction in cost after surgery from a mean preoperative annual direct cost of $1500–$2700 to a postoperative cost of $600–$1200 per patient [3, 44, 45, 46]. Surgery has also been shown to reduce the indirect costs of CRS. For example, while two‐thirds of patients with CRS experience an impairment in productivity and activity prior to sinus surgery, potentially attributed to sleep and psychological problems related to their disease, nearly three‐quarters reported improved productivity following surgery [2, 47].

Given that costs for surgery can vary depending on institutional, geographic, and insurance‐related variables, a single variable sensitivity analysis was performed in this study. It was found that doubling the cost of ESS resulted in a modest increase in the ICER result to $6857.59 per QALY, well below the cost‐effective threshold of $50,000 per QALY. This analysis thus highlights that the key driver of the cost‐effectiveness of ESS in this cohort is the significant, sustained improvement in quality of life that patients experience following sinus surgery.

An important limitation of the study is that assignment of patients to the surgical or medical cohort was based on self‐selection and not randomization. However, as stated previously, given that the key driver of this cost utility analysis was the substantial and sustained improvement in quality of life with sinus surgery as compared to medical management, it would be unlikely for the overall outcome of this model to change with randomization of patients. The high propensity for patients with nasal polyps to elect to undergo surgery meant that the sample size of patients with nasal polyps was limited and thus not able to be independently evaluated. Furthermore, because the majority of patients were treated prior to the introduction of biologic therapy for CRS, this variable is not accounted for in the economic model. The inclusion of biologic therapy in such an analysis is complicated by the lack of consensus with respect to upfront versus secondary treatment in CRS patients. Further data are needed regarding outcomes in these specific scenarios in order to accurately incorporate the effects of biologics on the cost‐effectiveness of various treatment algorithms for CRS. Nevertheless, prior CUAs have evaluated the cost‐effectiveness of upfront dupilumab compared to primary ESS for CRS patients with nasal polyps and found surgery to be more cost‐effective in a primary setting [13, 48]. Because all patients were enrolled at an academic tertiary care medical center, the study results may not be applicable to the general population. Comparison of CRS patient cohorts treated in a variety of practice types and geographic locations consistently shows symptomatic improvements following sinus surgery, although there may be variation in the magnitude of this improvement [49]. Further study is needed to understand whether the quality of life outcomes experienced in this academic cohort, and the cost effectiveness associated with those outcomes, are generalizable to the broader population.

## 5 Conclusion

Cost utility analysis shows ESS to be a cost‐effective intervention compared to medical therapy alone for the management of patients with CRS. This analysis improves upon prior economic models by including long‐term (5‐year) surgical outcomes data and by utilizing prospectively collected utility data on patients medically managed by otolaryngologists.

## Conflicts of Interest

The authors declare no conflicts of interest.

## Data Availability

The data that support the findings of this study are available from the corresponding author upon reasonable request.
